# Exercise modulation of BDNF/TrkB signaling in Parkinson’s disease: an evidence-calibrated review of neuroprotective mechanisms, biomarker limitations, and translational gaps

**DOI:** 10.3389/fneur.2026.1860227

**Published:** 2026-06-25

**Authors:** Chuan Chen, Kaihua Liu, Meng Xiang, Xueqin Zhang

**Affiliations:** 1 Jishou University, Jishou, China; 2 School of Physical Education and Arts, Hunan University of Medicine, Huaihua, China; 3 Changsha Preschool Education College, Changsha, China

**Keywords:** BDNF, exercise, neuroprotection, Parkinson’s disease, synaptic plasticity, TrkB

## Abstract

Parkinson’s disease (PD) is a progressive neurodegenerative disorder characterized by dopaminergic neuronal loss and persistent motor and non-motor impairment. Exercise has emerged as a promising and helpful adjunctive strategy for PD because it improves functional outcomes and may engage biological processes related to neuroprotection and neuroplasticity. Among these mechanisms, brain-derived neurotrophic factor (BDNF) and tropomyosin receptor kinase B (TrkB) signaling have received increasing attention. This review summarizes pathological alterations of BDNF/TrkB signaling in PD and evaluates how exercise may influence this system to support neuronal survival, synaptic plasticity, and microenvironmental homeostasis. Evidence from animal studies suggests that the phosphoinositide 3-kinase/Akt (PI3K/Akt) and mitogen-activated protein kinase/extracellular signal-regulated kinase (MAPK/ERK) pathways are mainly supported by preclinical PD exercise studies that assessed dopaminergic preservation, downstream pathway activation, and behavioral recovery. In contrast, phospholipase Cγ (PLCγ)-related signaling, TrkB isoform-specific regulation, astrocyte-dependent mechanisms, and trophic trafficking remain less clearly defined. This review also emphasizes key translational gaps, including peripheral BDNF biomarker limitations, the mismatch between human biomarker studies and animal mechanistic studies, and the need to match exercise prescriptions with specific BDNF/TrkB-related mechanisms.

## 1 Introduction

Parkinson’s disease (PD) is a progressive neurodegenerative disorder characterized primarily by degeneration of dopaminergic neurons in the substantia nigra pars compacta, depletion of striatal dopamine, and accumulation of pathological α-synuclein (1–4). These pathological changes disrupt basal ganglia circuit function and contribute to a broad spectrum of motor and non-motor manifestations, including bradykinesia, rigidity, tremor, gait disturbance, cognitive impairment, sleep disorders, autonomic dysfunction, and affective symptoms (5). Although current pharmacological treatments, particularly levodopa and dopamine agonists, provide substantial symptomatic benefit, they have not been shown to halt the underlying progression of PD and are often accompanied by long-term complications (6–9). In the present review, disease progression refers to the continued deterioration of nigrostriatal dopaminergic integrity, accumulation of disease-related pathology, and gradual worsening of motor and non-motor disability over time (10–12). This limitation of symptomatic pharmacotherapy has prompted increasing interest in helpful adjunctive strategies that may influence disease-relevant biological processes rather than motor symptoms alone.

Exercise has emerged as a clinically meaningful non-pharmacological intervention for PD because it is relatively safe, scalable, and compatible with pharmacological treatment, neuromodulation, and multidisciplinary rehabilitation (13–15). Structured exercise can improve gait, balance, mobility, postural control, muscle strength, quality of life, and selected non-motor symptoms (16–21). It may also contribute to broader PD management by supporting cardiovascular health, cognitive function, mood, sleep, fatigue, constipation, bone health, and dopaminergic system optimization (22–29). Therefore, exercise should not be viewed merely as general physical conditioning, but as a helpful adjunctive intervention with potential biological and functional relevance to PD.

The biological rationale for exercise in PD has developed from foundational work on use-dependent and time-dependent plasticity. Early animal studies showed that forced use, treadmill training, and enriched motor activity could improve motor performance, modify dopaminergic signaling, and help preserve remaining viable dopaminergic neurons in PD-relevant models (30–32). Human neurophysiological and neuroimaging studies further suggested that exercise can influence corticomotor excitability and metabolic network activity in patients with PD (33–35). These findings helped shift the field from viewing exercise as symptomatic training toward viewing it as an activity-dependent stimulus capable of engaging neural plasticity. In broader neurorehabilitation research, repeated activity has also been linked to neurotrophic factor expression, synaptogenesis, presynaptic and postsynaptic modulation, glucose utilization, immune regulation, angiogenesis, oxidative stress control, calcium homeostasis, and inflammation (36–40).

Among the molecular systems implicated in PD-related neuroprotection and neuroplasticity, brain-derived neurotrophic factor (BDNF)/tropomyosin receptor kinase B (TrkB)-mediated signaling has attracted considerable attention (41–43). BDNF is a major neurotrophin involved in neuronal survival, synaptic maintenance, dendritic remodeling, and activity-dependent plasticity, whereas TrkB is the principal receptor through which mature BDNF activates intracellular signaling cascades (44–48). In the nigrostriatal system, dopaminergic neurons are exposed to high metabolic demand, oxidative stress, long axonal projections, and complex glia–neuron interactions (49–52). Under these conditions, impairment of BDNF/TrkB signaling may reduce adaptive plasticity and increase neuronal vulnerability (53–55). However, this relationship should be interpreted cautiously. Abnormal BDNF/TrkB signaling should not be viewed as a single determinant of PD progression, but rather as one component of a broader pathological network involving mitochondrial dysfunction, oxidative stress, neuroinflammation, impaired protein clearance, synaptic instability, and axonal degeneration (43, 55–57).

Exercise may influence this network through several partly overlapping mechanisms. Preclinical studies using toxin-induced and genetic PD models suggest that exercise may engage biological processes related to neuronal survival, synaptic remodeling, mitochondrial regulation, antioxidant defense, and inflammatory control (31, 32, 58–60). Within this broader framework, BDNF/TrkB signaling is considered a biologically plausible link between repeated physical activity and adaptive neural responses (61–63). In particular, exercise-related effects on dopaminergic integrity, synaptic protein expression, oxidative stress, inflammatory responses, and behavioral recovery are relevant to the three major mechanistic domains emphasized in this review: neuronal survival, synaptic plasticity, and microenvironmental homeostasis. This relevance to human PD is important because lifelong physical activity has been associated with reduced PD risk, whereas reduced physical activity may appear as an early presymptomatic feature of PD (31, 58, 64–66).

Nevertheless, the current evidence remains uneven and requires careful interpretation. In human studies, BDNF is commonly measured in serum or plasma because peripheral blood sampling is accessible and minimally invasive (67–69). However, peripheral BDNF is affected by platelet release, sample processing, inflammatory status, medication exposure, circadian rhythm, age, sex, and systemic responses to exercise (70–73). Therefore, serum or plasma BDNF should be regarded as an indirect and supportive biomarker of exercise responsiveness rather than a direct readout of central BDNF/TrkB activation in the brain. In contrast, animal models provide more direct access to nigral and striatal tissue and allow assessment of TrkB phosphorylation, downstream signaling pathways, dopaminergic neuron survival, tyrosine hydroxylase expression, and behavioral recovery (43, 74–76). Even so, commonly used models such as 6-hydroxydopamine and 1-methyl-4-phenyl-1,2,3,6-tetrahydropyridine do not fully reproduce the progressive and multisystem nature of human PD (77–79).

In this context, the present review aims to provide an evidence-calibrated synthesis of the relationship among exercise, BDNF/TrkB signaling, and PD-related neuroprotection. Rather than simply reiterating that exercise may increase BDNF, this review focuses on three interrelated issues. First, it evaluates neuroprotective mechanisms through which exercise may support neuronal survival, synaptic plasticity, and microenvironmental homeostasis. Second, it discusses biomarker limitations, especially why peripheral BDNF cannot be interpreted as a direct surrogate for central BDNF/TrkB activity. Third, it identifies translational gaps, including the mismatch between human biomarker studies and animal mechanistic studies, the limited causal evidence for specific downstream pathways, unresolved TrkB isoform-specific and astrocyte-related mechanisms, and the need for exercise prescription–mechanism matching. This review first summarizes the biological architecture of BDNF/TrkB signaling, then discusses pathological alterations of this system in PD, evaluates exercise-related evidence from human and animal studies, and finally outlines biomarker limitations and translational gaps that should guide future mechanism-informed exercise research.

## 2 Methods and scope of this narrative review

This article was designed as a narrative review rather than a systematic review or meta-analysis. Therefore, no formal Preferred Reporting Items for Systematic Reviews and Meta-Analyses screening procedure, quantitative synthesis, risk-of-bias assessment, or PROSPERO registration was performed. The purpose of this review was to provide an evidence-calibrated synthesis of current knowledge regarding exercise, BDNF/TrkB signaling, and PD, with particular attention to the distinction between clinical observations, preclinical mechanistic evidence, and emerging hypotheses that require further validation.

Relevant peer-reviewed studies were identified from literature addressing PD pathology, exercise rehabilitation, PA, BDNF/TrkB biology, neurotrophic signaling, PD animal models, and exercise-induced neuroplasticity. The literature considered in this review included clinical studies, RCTs, systematic reviews, meta-analyses, foundational neurotrophin studies, and mechanistic animal studies. Search terms included combinations of “Parkinson’s disease,” “exercise,” “physical activity,” “BDNF,” “TrkB,” “neuroprotection,” “neuroplasticity,” “6-OHDA,” “MPTP,” “astrocyte,” “PI3K/Akt,” “MAPK/ERK,” “CREB,” and “TH.” Priority was given to studies that directly examined exercise-related functional outcomes, BDNF/TrkB-related molecular changes, dopaminergic integrity, synaptic plasticity, glial or inflammatory responses, or downstream signaling pathways relevant to PD.

Because evidence in this field comes from heterogeneous sources, this review distinguishes between human clinical evidence and animal mechanistic evidence wherever possible. Human studies were used primarily to evaluate exercise feasibility, clinical relevance, functional outcomes, peripheral biomarker responses, and associations with motor or non-motor symptoms. However, they generally cannot directly determine whether exercise activates central BDNF/TrkB signaling in the SNpc, striatum, or other PD-relevant brain regions. Preclinical animal studies were used primarily to evaluate mechanistic relationships, including changes in nigrostriatal BDNF/TrkB signaling, TrkB phosphorylation, PI3K/Akt and MAPK/ERK signaling, TH expression, dopaminergic neuron survival, synaptic proteins, glial activation, oxidative stress, inflammation, and behavioral recovery.

To reduce overinterpretation, this review differentiates between mechanisms with comparatively direct experimental support and mechanisms that remain biologically plausible but less directly validated. The PI3K/Akt–GSK3β and MAPK/ERK–CREB pathways are discussed as downstream axes with comparatively more direct preclinical support because PD exercise studies have examined these pathways together with TH preservation, apoptosis-related markers, synaptic protein expression, and behavioral recovery. In contrast, PLCγ-related signaling, trophic trafficking, TrkB isoform-specific regulation, and astrocyte-dependent modulation are treated as emerging directions because they have rarely been tested directly using pathway-specific, isoform-specific, or cell type-specific approaches in PD exercise paradigms. This evidence hierarchy was used to clarify which conclusions are clinically supported, which are mechanistically supported in animal models, and which remain translational gaps.

Finally, the mechanistic synthesis focuses on three domains emphasized in the abstract: neuronal survival, synaptic plasticity, and microenvironmental homeostasis. Neuronal survival refers mainly to the preservation of vulnerable dopaminergic neurons or dopaminergic terminal integrity under PD-related stress. Synaptic plasticity refers to exercise-related changes in synaptic maintenance, activity-dependent remodeling, and circuit adaptation. Microenvironmental homeostasis refers to the regulation of oxidative stress, mitochondrial function, inflammatory activity, glial responses, and trophic support within PD-relevant neural environments.

## 3 Biological architecture of BDNF/TrkB signaling

### 3.1 Ligand processing: proBDNF, mature BDNF, and functional direction of signaling

Brain-derived neurotrophic factor (BDNF) is a major neurotrophin involved in neuronal survival, differentiation, synaptic maintenance, dendritic remodeling, and activity-dependent plasticity (45, 80–82). BDNF is initially synthesized as precursor BDNF (proBDNF), which can be cleaved intracellularly or extracellularly to generate mature BDNF (mBDNF) (83). These two molecular forms are not functionally equivalent. In general, proBDNF preferentially interacts with the p75 neurotrophin receptor (p75NTR)–sortilin complex and is more often associated with synaptic weakening, structural regression, and pro-apoptotic signaling, whereas mBDNF primarily activates tropomyosin receptor kinase B (TrkB) to support neuronal survival and plasticity (84–86).

This distinction is important because the biological meaning of BDNF cannot be inferred from total BDNF abundance alone. A shift from mBDNF–TrkB signaling toward proBDNF–p75NTR/sortilin signaling may indicate a less supportive trophic environment, even when total BDNF measurements appear unchanged (84, 86, 87). Therefore, studies that measure only total BDNF, particularly in peripheral blood, may not fully capture the functional direction of neurotrophic signaling.

### 3.2 TrkB-FL, TrkB.T1, and canonical downstream pathways

Among BDNF receptors, full-length TrkB (TrkB-FL) is the principal signaling receptor mediating the neuroprotective and plasticity-related effects of mature BDNF (88–91). Binding of mBDNF to TrkB-FL induces receptor dimerization and autophosphorylation, which subsequently activates several intracellular signaling cascades (92, 93). The phosphoinositide 3-kinase (PI3K)/Akt pathway is mainly associated with cell survival, mitochondrial protection, and anti-apoptotic regulation (94, 95). The mitogen-activated protein kinase/extracellular signal-regulated kinase (MAPK/ERK) pathway contributes to transcriptional regulation, synaptic remodeling, and long-term plasticity (96–99). The phospholipase Cγ (PLCγ) pathway participates in intracellular Ca^2^+ signaling and activity-dependent modulation (100–103).

The functional output of BDNF/TrkB signaling is also influenced by receptor isoform composition. TrkB. T1 lacks the intracellular tyrosine kinase domain and therefore does not initiate classical TrkB-FL kinase signaling in the same manner (104–106). It is enriched in astrocytes and may participate in Ca^2^ + regulation, cytoskeletal organization, extracellular homeostasis, and local trophic modulation (104, 105, 107, 108). However, compared with the canonical mBDNF–TrkB-FL axis, the contribution of TrkB. T1 to PD-related neurodegeneration and exercise-induced neuroprotection remains less clearly defined.

### 3.3 Signaling endosomes and long-range trophic communication

BDNF/TrkB signaling is not limited to receptor activation at the cell surface. After ligand binding, activated TrkB receptors can undergo internalization, meaning that receptor–ligand complexes are taken into the cell through endocytic mechanisms and incorporated into signaling endosomes (109). These signaling endosomes can be transported from distal neuronal compartments toward the soma, linking local trophic capture to nuclear transcriptional responses and long-term survival programs (110–113).

This process is particularly relevant for nigrostriatal dopaminergic neurons because they have long and highly arborized projections from the SNpc to the striatum (52). Anatomical reconstruction studies have reported that single rat nigrostriatal dopaminergic neurons can form widely distributed striatal axonal arborizations, with total axonal lengths of approximately 140,000–780,000 μm (114). In PD, axonal injury, cytoskeletal disruption, mitochondrial dysfunction, and impaired intracellular transport may weaken TrkB internalization, endosomal sorting, retrograde transport, or receptor recycling, thereby reducing long-range trophic communication (115–117).

### 3.4 Astrocyte-related trophic regulation in the nigrostriatal microenvironment

Astrocytes have traditionally received less attention than neurons and microglia in PD research, but their importance has become increasingly recognized. This shift reflects a broader understanding that PD-related neurodegeneration occurs within a multicellular microenvironment rather than within dopaminergic neurons alone. Astrocytes are present throughout the striatum and substantia nigra and contribute to extracellular glutamate and potassium homeostasis, metabolic support, antioxidant defense, inflammatory modulation, blood–brain barrier regulation, and synaptic stability (118–120). Single-cell and cell-type studies of the striatum have also confirmed that astrocytes represent an important non-neuronal component of the striatal cellular landscape, whereas medium spiny neurons constitute the major neuronal population of the striatum (121, 122).

Astrocytes may influence BDNF availability through synthesis, release, uptake, extracellular processing, and microenvironmental regulation (71, 123–125). Because TrkB. T1 is enriched in astrocytes, astrocyte-related BDNF/TrkB signaling may also affect Ca^2^+ dynamics, cytoskeletal remodeling, extracellular homeostasis, and local trophic buffering (108, 126). Nevertheless, direct evidence that exercise specifically modifies astrocytic TrkB. T1 signaling in PD remains limited; this mechanism should therefore be presented as an emerging direction rather than an established core pathway.

### 3.5 Regional vulnerability and trophic reserve in PD-relevant circuits

BDNF/TrkB signaling shows marked regional heterogeneity across the brain (54, 61, 89, 126, 127). The cortex and hippocampus are major sources of BDNF, and substantial trophic support can be delivered to the striatum through corticostriatal projections (128). By contrast, dopaminergic neurons in the substantia nigra pars compacta appear to possess relatively limited intrinsic trophic reserve and may depend more strongly on exogenous trophic input, axonal transport, and glial support (51, 129, 130).

**Figure 1:**
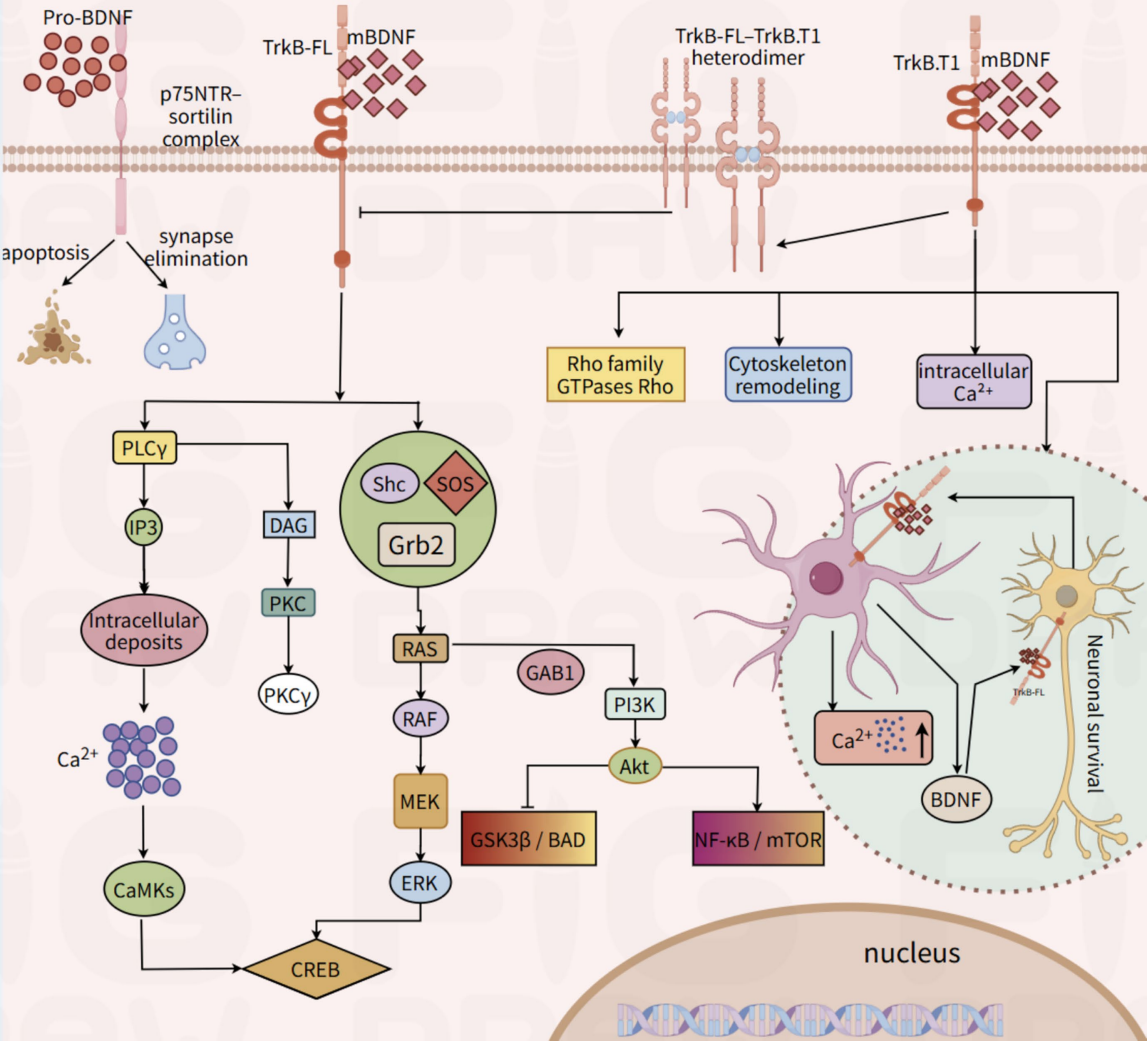
Biological architecture of BDNF/TrkB signaling and astrocyte-related trophic regulation.

In this review, “limited trophic reserve” refers to the constrained capacity of vulnerable nigrostriatal dopaminergic neurons to maintain sufficient neurotrophic support under oxidative stress, mitochondrial dysfunction, axonal injury, and inflammatory burden. Within this context, impaired ligand processing, weakened TrkB-FL signaling, disrupted retrograde trophic trafficking, and altered receptor isoform regulation may contribute to increased nigrostriatal vulnerability (43, 106, 131, 132). The major components of BDNF/TrkB signaling discussed in this section are summarized in Figure 1.

BDNF is synthesized as proBDNF and subsequently cleaved into mBDNF. proBDNF preferentially binds the p75NTR–sortilin complex and is generally associated with synaptic weakening and pro-apoptotic signaling, whereas mBDNF activates TrkB-FL to engage PI3K/Akt, MAPK/ERK, and PLCγ pathways involved in neuronal survival, synaptic plasticity, and Ca^2^+-dependent regulation (84, 86, 133). BDNF/TrkB signaling also involves receptor internalization and signaling endosome trafficking, which support long-range trophic communication from distal neuronal compartments to the soma (47, 109, 134). Astrocyte-enriched TrkB. T1 may contribute to Ca^2^+ regulation, extracellular homeostasis, and local trophic buffering (104, 108, 135). This figure was created by the authors using the Hiplot Scientific Research Drawing Platform. No AI-generated image content was used.

## 4 Alterations of BDNF/TrkB signaling in Parkinson’s disease

Alterations of BDNF/TrkB signaling in PD should not be understood simply as reduced neurotrophic factor abundance. Current evidence suggests a broader disturbance of trophic homeostasis involving ligand processing, receptor composition, intracellular signaling, long-range trophic communication, and glia-related regulation (43, 117, 136–138). Within the nigrostriatal system, where dopaminergic neurons are exposed to high metabolic demand, oxidative stress, axonal vulnerability, and inflammatory burden, such disturbances may reduce adaptive plasticity and increase susceptibility to degeneration (52, 139–142). However, BDNF/TrkB dysfunction should be interpreted as one component of a larger pathological network rather than as a single driver of PD progression.

### 4.1 Evidence from human Parkinson’s disease studies

Postmortem human PD studies support the presence of impaired neurotrophic signaling in vulnerable nigrostriatal regions. Reduced BDNF mRNA or protein levels have been reported in the SNpc, and some studies have also described downregulation of TrkB-FL together with alterations in synaptic markers (43, 90, 143). These findings suggest that neurotrophic insufficiency may be involved in the cellular environment of human PD. However, postmortem evidence usually reflects advanced disease and cannot determine whether BDNF/TrkB impairment is a cause, consequence, or compensatory response to dopaminergic degeneration.

Peripheral studies have also reported altered BDNF levels in patients with PD. Serum or plasma BDNF levels are often lower in patients with PD than in healthy controls, and some studies have linked peripheral BDNF to non-motor symptoms, disease severity, or functional status (144–146). However, these findings are heterogeneous because peripheral BDNF is influenced by platelet release, blood processing procedures, medication exposure, inflammatory state, circadian variation, age, sex, and PA level (147–149). Therefore, peripheral BDNF can provide supportive information about systemic or exercise-related responsiveness, but it should not be treated as a direct surrogate for central BDNF/TrkB activity.

At the ligand level, total BDNF alone cannot define the functional direction of neurotrophic signaling. A shift toward proBDNF–p75NTR/sortilin signaling may favor synaptic weakening or pro-apoptotic responses, whereas mBDNF–TrkB-FL signaling generally supports neuronal survival and adaptive synaptic remodeling (48, 150, 151). This distinction is important because total BDNF measurements, especially in peripheral blood, may not reflect the balance between vulnerability and adaptive trophic signaling in PD.

### 4.2 Evidence from animal models

Animal models provide more direct access to nigral and striatal tissue and therefore allow mechanistic assessment of BDNF/TrkB-related changes under PD-like pathology. The 6-OHDA and MPTP models are among the most widely used toxin-induced models in PD research (152–156). The 6-OHDA model is commonly used to assess selective catecholaminergic degeneration, striatal TH depletion, rotational behavior, and neuroprotective interventions (157–159). The MPTP model reproduces mitochondrial complex I-related dopaminergic toxicity and is frequently used to evaluate motor impairment, nigral neuronal survival, striatal DA depletion, oxidative stress, and neurotrophic signaling responses (160–164).

The major advantage of these models is experimental control. Researchers can vary lesion timing, lesion severity, age, baseline fitness, exercise onset, exercise modality, training intensity, training duration, and tissue sampling time (165, 166). They also allow parallel assessment of behavior and brain-level outcomes, including TH expression, dopaminergic neuron survival, BDNF/TrkB signaling, oxidative stress, inflammatory responses, synaptic proteins, and apoptosis-related markers (167–169). These features make animal models useful for testing whether exercise can attenuate toxin-induced dopaminergic injury and engage trophic or plasticity-related mechanisms.

In toxin-induced PD models, nigral or striatal BDNF/TrkB signaling is often reduced during dopaminergic injury, together with decreased TH expression, impaired synaptic protein expression, mitochondrial dysfunction, oxidative stress, and increased apoptotic signaling (42, 43, 117, 137, 170). Reduced striatal TH immunoreactivity in 6-OHDA or MPTP models is commonly interpreted as evidence of nigrostriatal terminal loss or dysfunction, whereas partial preservation of TH after intervention suggests dopaminergic protection or functional recovery (171, 172).

Genetic and α-synuclein-related models provide complementary information because they may better capture selected features of protein aggregation, axonal transport defects, synaptic instability, and neuroinflammation (43, 173–175). However, direct evidence linking exercise, BDNF/TrkB signaling, and disease modification in these models remains less extensive than evidence from toxin-induced paradigms. Therefore, animal models support mechanistic plausibility, but their findings should not be directly translated into claims of disease modification in human PD.

### 4.3 Receptor-level and downstream signaling disturbances

Trophic failure in PD may involve not only reduced ligand availability but also impaired utilization of available trophic signals. At the receptor level, reductions in TrkB-FL or changes in the balance between TrkB-FL and TrkB. T1 may decrease the efficiency with which mBDNF is converted into pro-survival and pro-plasticity responses (54, 90, 91, 106, 176). In addition, α-synuclein pathology and axonal transport defects may interfere with TrkB internalization, receptor trafficking, degradation, and retrograde signaling, thereby weakening long-range trophic communication (43, 177–179).

Downstream of receptor activation, reduced PI3K/Akt signaling may weaken anti-apoptotic regulation and mitochondrial protection, whereas disruption of MAPK/ERK–CREB signaling may impair transcriptional programs required for synaptic maintenance and activity-dependent plasticity (180). PLCγ-dependent signaling may also become dysregulated, with potential consequences for intracellular Ca^2^+ homeostasis and trophic responsiveness (100, 103, 181, 182). These abnormalities are not specific to BDNF/TrkB alone, but their convergence with impaired neurotrophic support provides a plausible mechanism through which trophic dysfunction may contribute to cellular vulnerability in PD.

### 4.4 Glial and microenvironmental regulation

PD-related trophic disturbance also occurs within a broader microenvironmental context. Chronic oxidative stress, mitochondrial dysfunction, and persistent neuroinflammation can destabilize BDNF/TrkB signaling by reducing trophic support capacity and impairing signal integration (42, 183–185). Astrocytes are relevant because they regulate extracellular homeostasis, provide metabolic and antioxidant support, modulate inflammatory signaling, and influence synaptic stability (186–188).

The specific contribution of astrocyte-dependent BDNF/TrkB regulation in PD remains incompletely defined. TrkB. T1 is enriched in astrocytes and may influence Ca^2^+ dynamics, cytoskeletal remodeling, extracellular homeostasis, and trophic buffering (104, 105, 108, 135, 189). However, compared with the neuronal mBDNF–TrkB-FL axis, the role of astrocytic TrkB. T1 in PD and exercise-related neuroprotection remains an emerging field rather than an established central pathway.

Taken together, BDNF/TrkB alterations in PD can be understood across four interconnected levels: impaired ligand processing and availability, altered receptor composition, reduced downstream signaling efficiency, and disrupted trophic support within the nigrostriatal microenvironment. This multi-level disturbance provides a biological basis for examining whether exercise can engage BDNF/TrkB-related mechanisms to support neuronal survival, synaptic stability, and functional adaptation in PD.

## 5 Exercise and BDNF/TrkB-related adaptations in Parkinson’s disease

Exercise has emerged as a helpful adjunctive strategy for PD because it acts across functional, systemic, and neural levels rather than targeting dopaminergic replacement alone (33, 190). Regular exercise can influence cardiorespiratory function, skeletal muscle metabolism, motor learning, sensorimotor integration, endocrine responses, and neural plasticity (191–194). This multi-level profile is relevant to PD, which involves not only nigrostriatal dopaminergic degeneration but also impairments in gait, balance, cognition, sleep, affective regulation, autonomic function, fatigue, and daily activity (195, 196). Because the strength of evidence differs across study types, this section separates human clinical evidence from animal mechanistic evidence.

### 5.1 Human clinical evidence

Clinical studies generally support the functional value of structured exercise in patients with PD. Aerobic training, resistance exercise, balance training, Tai Chi, dance, treadmill training, cycling, and multimodal rehabilitation have been reported to improve gait, postural stability, mobility, muscle strength, balance confidence, quality of life, and selected non-motor symptoms (21, 35, 197–201). These findings support exercise as a clinically useful adjunctive intervention, although the magnitude of benefit varies according to disease stage, symptom profile, medication state, intervention duration, supervision, adherence, and exercise modality.

For human PD, the strongest conclusion is functional rather than mechanistic. Improvements in gait speed, walking endurance, balance, motor severity, mood, sleep, or quality of life may reflect motor learning, cardiovascular conditioning, muscular adaptation, medication responsiveness, cortical plasticity, motivational factors, and systemic anti-inflammatory effects (16, 21, 144, 202–204). Therefore, functional improvement after exercise should not automatically be interpreted as evidence of central BDNF/TrkB activation.

Human studies most commonly rely on serum or plasma BDNF because direct assessment of central BDNF/TrkB activity is not feasible in routine clinical settings (205, 206). Some studies and meta-analyses report increased peripheral BDNF after exercise, sometimes alongside motor or non-motor improvement (65, 144, 159, 201, 207). However, peripheral BDNF is affected by platelet release, sample handling, inflammatory status, medication exposure, circadian variation, age, sex, baseline PA level, and acute exercise responses (70, 151, 208–211). Thus, peripheral BDNF should be interpreted as an indirect biomarker rather than direct evidence of central TrkB activation. Representative human studies, including their design, sample characteristics, exercise paradigms, BDNF outcomes, and functional endpoints, are summarized in Table 1.

**Table 1:** Representative evidence linking exercise interventions to BDNF/TrkB-related changes and functional or pathological outcomes in PD.

Study/citation	Evidence type	Population or model	Exercise paradigm	BDNF/TrkB-related evidence	Main functional or pathological outcome	Key limitation
Kaagman et al. (269)	Systematic review and meta-analysis of RCTs	Patients with PD; 5 RCTs; *N* = 216; H&Y mean ± SD: NR	Motorized treadmill, Speedflex machine, rowing machine, and other exercise/physical therapy interventions	Exercise significantly increased serum BDNF; pooled effect: SMD = 1.20, 95% CI 0.53 to 1.87, *p* = 0.0004, *I*^2^ = 77%	Across the included RCTs, exercise interventions were associated with increased serum BDNF, with the pooled BDNF effect reported as SMD = 1.20, 95% CI 0.53 to 1.87, *p* = 0.0004, *I*^2^ = 77%. Clinical outcomes such as UPDRS, UPDRS-III, 6MWT, and BBS were reported across the included studies, but the main quantified meta-analytic result directly relevant to this table was the serum BDNF effect	High heterogeneity was present in the pooled BDNF analysis; included interventions and clinical endpoints were heterogeneous; serum BDNF is an indirect peripheral biomarker and cannot establish central BDNF/TrkB activation
Frazzitta et al. (270)	Randomized clinical rehabilitation study	Patients with early-stage PD treated with rasagiline; *N* = 30	Intensive rehabilitation for 28 days, 3 h/day, including aerobic exercise	Serum BDNF increased after 10 days and remained elevated throughout treatment; reported BDNF effect size = 1.1	UPDRS-III improved after rehabilitation; UPDRS II, total UPDRS, BBS, and 6MWT also improved	Multimodal rehabilitation design; although serum BDNF and clinical outcomes improved, the isolated contribution of aerobic exercise or BDNF/TrkB signaling cannot be determined
Hirsch et al. (225)	Systematic review and meta-analysis	Ambulatory patients with idiopathic PD; 2 RCTs and 4 pre-experimental studies; total *N* = 100; H&Y ≤ 3	Exercise and rehabilitation interventions across included studies	All included studies reported increased blood BDNF after exercise; pooled RCT effect for BDNF: SMD = 2.06, 95% CI 1.36 to 2.76	UPDRS-III improved in pooled RCT analysis; MD = −5.53, 95% CI −10.42 to −0.64	Small number of studies; preliminary evidence with limited methodological quality
6-OHDA model studies (31, 159)	Preclinical animal evidence	6-OHDA-induced PD model	Treadmill training, forced-use exercise, or other structured exercise paradigms	Exercise was associated with increased BDNF/TrkB-related signaling or attenuation of dopaminergic injury-related molecular changes	Preservation of striatal TH or SNpc TH-positive neurons and improvement in rotational behavior or motor performance	Lesion severity, exercise timing, and molecular endpoints differ across studies; findings cannot be directly translated to human disease modification
MPTP model studies (60, 66)	Preclinical animal evidence	MPTP-induced PD model	Treadmill training, voluntary running, or aerobic exercise paradigms	Exercise was associated with BDNF-related signaling changes together with reduced oxidative stress, inflammatory responses, or apoptosis-related markers	Improved motor performance and partial preservation of dopaminergic integrity	Acute toxin-induced injury does not fully reproduce progressive human PD
α-synuclein-related or genetic model studies (214, 215)	Preclinical animal evidence	α-synuclein-related or genetic PD-relevant models	Voluntary wheel running, enriched environment, or structured exercise paradigms	Exercise-related BDNF/TrkB evidence is less extensive than in toxin-induced models	May inform synaptic instability, protein pathology, or inflammatory regulation	Evidence remains limited and model-specific
Real et al. (271)	Mechanistic preclinical study	6-OHDA-induced rat model of PD	Exercise combined with BDNF receptor blockade	BDNF receptor blockade hindered the beneficial effects of exercise	Attenuated exercise-related protection in the PD model	Supports BDNF/TrkB-related causality in an experimental model, but does not establish central BDNF/TrkB-mediated disease modification in humans

BBS, Berg Balance Scale; BDNF, brain-derived neurotrophic factor; CI, confidence interval; H&Y, Hoehn and Yahr; MD, mean difference; MPTP, 1-methyl-4-phenyl-1,2,3,6-tetrahydropyridine; NR, not reported; PD, Parkinson’s disease; RCT, randomized controlled trial; SMD, standardized mean difference; TH, tyrosine hydroxylase; TrkB, tropomyosin receptor kinase B; 6MWT, 6-min walk test; 6-OHDA, 6-hydroxydopamine; UPDRS, Unified Parkinson’s Disease Rating Scale.

### 5.2 Evidence from animal models

Animal models provide more direct access to nigral and striatal tissue and therefore allow closer examination of exercise-related BDNF/TrkB signaling (212, 213). In MPTP, 6-OHDA, lipopolysaccharide, α-synuclein-related, and genetic PD models, treadmill training, voluntary wheel running, forced-use exercise, aerobic exercise, and enriched-environment paradigms have been reported to improve motor performance while partially preserving dopaminergic integrity. These effects are often accompanied by increased BDNF expression, enhanced TrkB-related signaling, TH preservation, improved synaptic protein levels, reduced oxidative stress, attenuated inflammatory responses, and decreased apoptotic signaling (31, 60, 66, 159, 214, 215).

The experimental advantage of animal models is that lesion timing, lesion severity, exercise onset, modality, intensity, duration, age, sex, baseline fitness, and tissue sampling can be controlled. In 6-OHDA models, exercise-related preservation of striatal TH is commonly interpreted as improved dopaminergic terminal integrity, whereas preservation of SNpc TH-positive neurons reflects protection of dopaminergic cell bodies (216, 217). In MPTP models, exercise-related improvement is often interpreted in relation to mitochondrial complex I-related toxicity, oxidative stress, inflammation, nigral DA neuron survival, striatal DA depletion, and neurotrophic signaling (31).

Importantly, several preclinical studies suggest that BDNF/TrkB signaling is not merely associated with exercise-induced benefit but may be functionally relevant. Pharmacological or genetic disruption of BDNF or TrkB signaling has been reported to attenuate exercise-related protection of dopaminergic neuron survival, synaptic integrity, or motor behavior (159, 218, 219). Such findings provide stronger mechanistic support than clinical biomarker studies alone. Nevertheless, direct causal studies remain limited, and the contribution of TrkB-FL, TrkB. T1, astrocyte-related signaling, trophic trafficking, and downstream pathway selectivity remains unresolved.

Taken together, human studies support the clinical usefulness of exercise in PD, whereas animal models provide stronger mechanistic evidence that exercise can engage BDNF/TrkB-related pathways under controlled pathological conditions. The defensible conclusion is not that exercise has been proven to modify PD progression through BDNF/TrkB signaling, but that exercise can improve functional outcomes in human PD and engage BDNF/TrkB-related mechanisms in experimental models. Representative clinical and preclinical evidence is summarized in Table 1.

## 6 Neuroprotective mechanisms linking exercise to BDNF/TrkB-related adaptation in PD

Exercise is unlikely to influence BDNF/TrkB signaling through a single linear pathway. Instead, it may engage a broader adaptive network involving neuronal survival, synaptic plasticity, mitochondrial regulation, oxidative stress control, inflammatory modulation, and glia–neuron communication (40, 220–224). The PI3K/Akt–GSK3β and MAPK/ERK–CREB axes have comparatively more direct preclinical support in PD exercise studies because they have been examined alongside TH preservation, apoptosis-related markers, synaptic protein expression, and behavioral recovery (225–227). In contrast, PLCγ-related signaling, trophic trafficking, TrkB isoform-specific regulation, and astrocyte-dependent modulation remain biologically plausible mechanisms, but they have rarely been tested directly with pathway-specific, isoform-specific, or cell type-specific approaches in PD exercise paradigms. Accordingly, this section organizes the evidence around three neuroprotective domains: neuronal survival, synaptic plasticity, and microenvironmental homeostasis.

### 6.1 Neuronal survival

Neuronal survival is central to PD-related neuroprotection because degeneration of SNpc dopaminergic neurons and loss of striatal dopaminergic terminals are core pathological features of the disease (26, 228, 229). In experimental PD models, dopaminergic neurons are vulnerable to mitochondrial dysfunction, oxidative stress, impaired axonal transport, calcium dysregulation, and apoptosis-related signaling (230–233). Within this context, the BDNF/TrkB–PI3K/Akt axis is one of the pathways with comparatively direct preclinical support, particularly when pathway activation is reported together with TH preservation, reduced apoptosis-related signaling, improved mitochondrial function, or behavioral recovery.

After mBDNF binds to TrkB-FL, PI3K can activate Akt, which regulates downstream targets involved in mitochondrial protection and apoptotic control, including GSK3β-related signaling (133). In PD-relevant models, this pathway is often evaluated together with Bcl-2, Bax, cytochrome c, caspases, oxidative stress indices, TH expression, and dopaminergic neuron survival (234–236). Exercise studies using 6-OHDA and MPTP models have reported improved motor performance accompanied by partial TH preservation, reduced apoptosis-related markers, improved mitochondrial function, and enhanced BDNF/TrkB-related signaling (31, 42, 218, 237).

These findings suggest that exercise can engage survival-related BDNF/TrkB mechanisms under controlled experimental conditions. However, exercise-related TH preservation or dopaminergic neuron protection in animal models should be interpreted as partial protection or functional recovery, not as proof of complete reversal of PD pathology or definitive disease modification in humans.

### 6.2 Synaptic plasticity

PD involves not only dopaminergic neuron loss but also synaptic dysfunction and maladaptive remodeling of basal ganglia–cortical circuits. Because exercise repeatedly recruits sensorimotor circuits, it is especially relevant to activity-dependent synaptic remodeling. BDNF/TrkB signaling can influence this process through the MAPK/ERK–CREB pathway, which regulates transcriptional programs related to synaptic maintenance, dendritic remodeling, neurotransmitter release, and long-term plasticity (15, 66, 238, 239).

Synapsin, synaptophysin, PSD-95, Arc, and BDNF itself are commonly used as synaptic or plasticity-related markers (240–243). In PD-relevant models, exercise has been reported to increase BDNF availability, enhance ERK and CREB phosphorylation, improve synaptic protein expression, and improve behavioral performance (32, 115, 207). These findings suggest that exercise may support functional adaptation not only by preserving dopaminergic neurons, but also by improving synaptic efficiency, motor learning, corticostriatal communication, and compensatory circuit recruitment.

Human evidence is more indirect. Improvements in gait, balance, mobility, postural control, and motor function after exercise are consistent with activity-dependent plasticity, but they do not by themselves identify BDNF/TrkB–MAPK/ERK–CREB signaling as the causal mechanism (144, 244). Therefore, synaptic plasticity provides an important conceptual bridge between clinical exercise benefit and preclinical BDNF/TrkB mechanisms, but direct human mechanistic validation remains limited.

### 6.3 Microenvironmental homeostasis

The potential neuroprotective effects of exercise in PD cannot be explained by neuronal signaling alone. PD-related degeneration occurs within a complex microenvironment involving oxidative stress, mitochondrial dysfunction, impaired autophagy–lysosome activity, chronic inflammation, glial activation, vascular-metabolic changes, and disrupted trophic support (245–248). BDNF/TrkB signaling intersects with these processes, but it should not be presented as the sole upstream controller of exercise-induced effects.

Exercise has been reported to improve antioxidant responses, reduce oxidative stress markers, support mitochondrial quality control, partially restore autophagy-related proteins, and attenuate chronic inflammatory or glial responses in PD-relevant models (249, 250). These changes may create a cellular environment more permissive for trophic signaling and synaptic stability. However, some of these effects may also involve parallel pathways, including Nrf2-related antioxidant defense, AMPK-related metabolic regulation, mitochondrial biogenesis, and systemic anti-inflammatory responses (251–254).

Astrocytes may contribute to this microenvironmental response because they regulate extracellular homeostasis, metabolic and antioxidant support, inflammatory signaling, and synaptic stability (119, 187, 255). Because TrkB. T1 is enriched in astrocytes, exercise-induced changes in astrocyte-related BDNF/TrkB signaling may influence Ca^2^+ dynamics, cytoskeletal organization, extracellular homeostasis, and local trophic buffering (107, 256). Nevertheless, direct evidence that exercise specifically regulates astrocytic TrkB. T1 in PD remains limited, so this mechanism should be presented as emerging rather than established.

### 6.4 Emerging mechanisms and translational gaps

Beyond PI3K/Akt and MAPK/ERK signaling, BDNF/TrkB activation may also engage PLCγ-related Ca^2^+ signaling, receptor internalization, signaling endosome trafficking, and isoform-specific regulation (90, 134, 257, 258). These mechanisms are relevant because dopaminergic neurons are sensitive to Ca^2^+ dysregulation, mitochondrial workload, axonal transport defects, and impaired trophic communication. In principle, exercise could influence these processes by increasing neuronal activity, synaptic demand, vascular-metabolic support, cytoskeletal stability, and glia–neuron communication (40, 215, 259, 260).

However, most exercise studies do not directly measure PLCγ activation, TrkB internalization, endosomal dynamics, receptor recycling, retrograde transport, or cell type-specific TrkB isoforms. Future studies should therefore combine exercise paradigms with isoform-specific assays, pathway inhibition, cell type-specific manipulation, spatial molecular techniques, and longitudinal behavioral assessment. Such approaches would help determine whether exercise-induced BDNF/TrkB changes reflect functionally meaningful trophic signaling rather than nonspecific protein expression changes.

**Figure 2:**
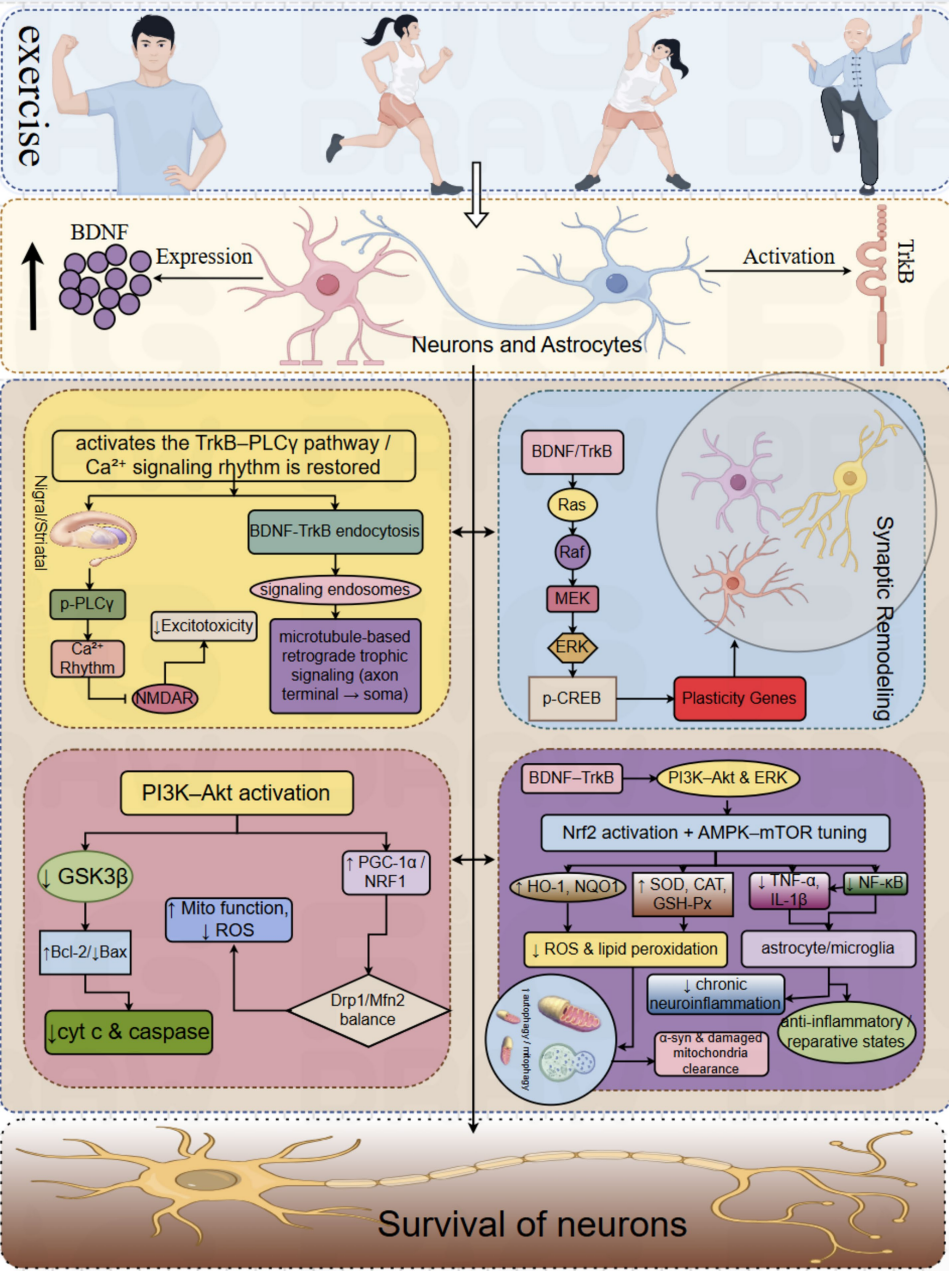
Evidence-calibrated framework linking exercise to BDNF/TrkB-related neuroprotective mechanisms in PD.

Taken together, exercise may support PD-related neuroprotection through three interrelated domains: neuronal survival, synaptic plasticity, and microenvironmental homeostasis. Among the pathways reviewed, PI3K/Akt–GSK3β and MAPK/ERK–CREB currently have the clearest preclinical support in PD exercise studies, particularly when pathway activation is reported together with TH preservation, reduced apoptosis-related signaling, improved synaptic markers, or behavioral recovery. By contrast, PLCγ signaling, trophic trafficking, TrkB isoform-specific regulation, and astrocyte-dependent modulation should be interpreted as emerging mechanisms requiring direct causal validation. This evidence-calibrated framework is summarized in Figure 2.

Exercise may influence PD-relevant outcomes through interconnected effects on neuronal survival, synaptic plasticity, and microenvironmental homeostasis. Evidence from animal models, including 6-OHDA, MPTP, lipopolysaccharide, α-synuclein-related, and genetic PD models, mainly supports exercise-related changes in BDNF/TrkB signaling through tissue-level outcomes such as TH preservation, downstream PI3K/Akt–GSK3β and MAPK/ERK–CREB activation, oxidative stress regulation, inflammatory modulation, and behavioral recovery (31, 115, 218). Human exercise studies support clinical improvement in gait, balance, mobility, motor function, and selected non-motor symptoms, but peripheral BDNF should be interpreted as an indirect biomarker rather than a direct measure of central TrkB activation (16, 20, 144). Resistance exercise is included because evidence suggests that resistance-based interventions may influence peripheral BDNF, although PD-specific mechanistic evidence remains more limited than that for aerobic or treadmill-based paradigms (207). Mechanisms shown as “supported mainly by preclinical PD exercise studies” include PI3K/Akt–GSK3β-related neuronal survival and MAPK/ERK–CREB-related synaptic plasticity. Mechanisms shown as “emerging or indirectly supported” include PLCγ-related signaling, trophic trafficking, TrkB isoform-specific regulation, and astrocyte-dependent modulation. BDNF, brain-derived neurotrophic factor; CREB, cAMP response element-binding protein; GSK3β, glycogen synthase kinase 3β; MAPK/ERK, mitogen-activated protein kinase/extracellular signal-regulated kinase; MPTP, 1-methyl-4-phenyl-1,2,3,6-tetrahydropyridine; PD, Parkinson’s disease; PI3K/Akt, phosphoinositide 3-kinase/Akt; PLCγ, phospholipase Cγ; TH, tyrosine hydroxylase; TrkB, tropomyosin receptor kinase B; 6-OHDA, 6-hydroxydopamine. This figure was created by the authors using the Hiplot Scientific Research Drawing Platform. No AI-generated image content was used.

## 7 Biomarker limitations and evidence constraints

Although current evidence supports a biologically plausible relationship among exercise, BDNF/TrkB signaling, and neuroprotection in PD, several limitations affect how strongly exercise-related BDNF/TrkB changes can be interpreted as evidence of central neuroprotection, disease modification, or clinically meaningful adaptation.

### 7.1 Peripheral and postmortem biomarker limitations

In human studies, serum or plasma BDNF is widely used because it is accessible, minimally invasive, and suitable for repeated sampling. However, peripheral BDNF cannot be treated as a direct surrogate for central BDNF/TrkB activation in the SNpc, striatum, cortex, or other PD-relevant brain regions. It is strongly influenced by platelet release, blood processing procedures, clotting time, centrifugation protocol, storage conditions, medication exposure, inflammatory state, circadian variation, age, sex, baseline PA level, and acute exercise responses. Therefore, a rise in serum or plasma BDNF after exercise may indicate systemic responsiveness, platelet-related release, vascular or metabolic adaptation, or peripheral neurotrophic signaling, but it does not directly demonstrate central TrkB activation or nigrostriatal neuroprotection.

Postmortem human PD studies provide valuable access to disease-relevant brain regions and can reveal reductions in BDNF, changes in TrkB expression, synaptic marker alterations, glial responses, and region-specific trophic abnormalities. However, postmortem evidence usually reflects late-stage or advanced disease and cannot determine whether trophic dysfunction is a cause, consequence, or compensatory response to PD pathology. Biomarker measurements may also be affected by postmortem interval, agonal state, tissue pH, fixation or freezing procedures, regional sampling differences, RNA or protein degradation, disease duration, and treatment history. Thus, postmortem findings are essential for confirming human brain abnormalities, but they cannot establish whether exercise restores central BDNF/TrkB signaling over time.

### 7.2 Limited mechanistic resolution

Many studies still interpret exercise-related benefit mainly through changes in total BDNF or total TrkB expression. However, the functional state of this system depends on proBDNF/mBDNF balance, TrkB-FL/TrkB. T1 isoform composition, receptor phosphorylation, receptor internalization, intracellular trafficking, downstream pathway activation, and cell type-specific signaling context. Therefore, increased total BDNF expression alone is insufficient to demonstrate effective central BDNF/TrkB-mediated neuroprotection.

This limitation is especially important in exercise studies. If an intervention increases total BDNF but does not show mBDNF availability, TrkB-FL phosphorylation, PI3K/Akt–GSK3β activation, MAPK/ERK–CREB signaling, TH preservation, synaptic marker improvement, or behavioral recovery, the mechanistic meaning of the BDNF change remains uncertain. Conversely, behavioral improvement alone cannot determine whether BDNF/TrkB signaling is necessary unless pathway-specific inhibition, receptor manipulation, or downstream signaling assessment is included. Future work should therefore move from total protein abundance toward pathway-resolved and cell type-resolved analysis.

### 7.3 Animal model constraints

Preclinical models such as 6-OHDA and MPTP provide useful platforms for studying nigrostriatal DA injury, TH loss, oxidative stress, mitochondrial dysfunction, apoptosis, inflammation, and BDNF/TrkB-related signaling. They also allow direct tissue analysis and experimental control over lesion timing, exercise onset, exercise intensity, training duration, and molecular endpoint selection.

However, these models do not fully reproduce the progressive, multisystem, and heterogeneous nature of human PD. Acute toxin-induced lesions are especially useful for testing neuroprotective mechanisms under controlled injury conditions, but they may not capture long-term α-synuclein propagation, gradual axonal degeneration, non-motor symptoms, medication-related complications, or patient-level heterogeneity. α-synuclein-related and genetic models may better represent selected disease-relevant processes, but exercise–BDNF/TrkB evidence in these models remains less extensive than evidence from toxin-induced paradigms. Therefore, mechanistic conclusions derived from animal models should not be directly translated to human PD without appropriate caution.

### 7.4 Exercise heterogeneity and causal limitations

Existing exercise studies differ substantially in modality, intensity, frequency, session duration, total intervention length, supervision, adherence, progression, medication state, disease stage, baseline fitness, and timing of biomarker collection. Aerobic training, resistance exercise, Tai Chi, dance, treadmill training, cycling, balance training, forced-use exercise, voluntary running, enriched environment, and multimodal rehabilitation may all produce benefits, but they are unlikely to engage identical biological mechanisms. This heterogeneity makes it difficult to define a stable dose–response relationship between exercise and BDNF/TrkB-related adaptation.

Causality remains another major limitation. In clinical studies, most evidence linking exercise, peripheral BDNF, and functional outcomes is associative. Even when exercise is accompanied by increased peripheral BDNF and improved motor or non-motor outcomes, this does not prove that BDNF/TrkB signaling directly mediates the benefit. Stronger causal evidence mainly comes from preclinical studies using BDNF/TrkB blockade, pathway inhibition, or genetic manipulation, but such studies remain limited and have not fully resolved receptor isoform-specific, astrocyte-dependent, circuit-specific, or trafficking-related mechanisms.

Taken together, current evidence supports BDNF/TrkB-related signaling as a candidate mechanism of exercise-induced neuroprotection and neuroplasticity in PD. However, the field has not yet reached the mechanistic precision required to determine which components of the BDNF/TrkB network are necessary, sufficient, or clinically predictive of durable exercise benefit.

## 8 Translational gaps and future directions

Future research should move from descriptive association toward mechanism-guided validation. The major translational gap is not whether exercise is clinically useful in PD, but whether exercise-induced changes in peripheral biomarkers, central BDNF/TrkB activity, and durable functional outcomes can be connected within the same experimental or clinical framework.

### 8.1 Improving biomarker validity

Future studies should move beyond total BDNF or total TrkB measurements and assess the functional state of the BDNF/TrkB system more precisely. This includes distinguishing proBDNF from mBDNF, measuring TrkB-FL and TrkB. T1 separately, assessing TrkB phosphorylation, and evaluating downstream pathway activation such as PI3K/Akt–GSK3β and MAPK/ERK–CREB signaling. These measurements would help determine whether exercise truly enhances trophic signaling capacity rather than simply altering total protein expression. Peripheral BDNF may remain useful as an accessible marker of systemic responsiveness, but it should not be interpreted alone. Future studies should standardize sample type, collection time, medication state, recent exercise exposure, processing protocol, storage conditions, assay platform, and platelet-related variables. In addition to serum and plasma, other compartments such as CSF, saliva, tears, and extracellular vesicles may provide complementary information, but their relationship with central BDNF/TrkB activity in PD still requires validation.

### 8.2 Matching exercise prescription with mechanisms

Future clinical and preclinical studies should report exercise modality, intensity, frequency, session duration, total intervention length, progression, adherence, supervision, disease stage, medication state, baseline fitness, and timing of biomarker collection. These variables are essential because aerobic training, resistance exercise, treadmill training, balance training, dance, Tai Chi, voluntary running, forced exercise, and multimodal rehabilitation may not engage identical biological mechanisms.

For example, aerobic and treadmill-based exercise may be more closely related to cardiorespiratory adaptation, motor-circuit recruitment, and BDNF-related plasticity, whereas resistance exercise may involve muscle-derived factors, endocrine responses, strength adaptation, and systemic neurotrophic changes. Multimodal rehabilitation, including the work by Frazzitta and colleagues, is clinically valuable because it can improve motor and functional outcomes while increasing serum BDNF, but it can be difficult to isolate which component drives biomarker change. Therefore, future studies should move from asking whether “exercise” increases BDNF to asking which exercise modality, dose, and timing best engage specific BDNF/TrkB-related mechanisms.

### 8.3 Strengthening causal and translational designs

Stronger causal designs are needed to determine whether BDNF/TrkB signaling is necessary for exercise-induced benefit. In animal studies, exercise paradigms should be combined with pathway-specific inhibition, TrkB isoform-selective manipulation, astrocyte-targeted interventions, and longitudinal assessment of dopaminergic integrity. These approaches could clarify whether PI3K/Akt–GSK3β, MAPK/ERK–CREB, PLCγ signaling, trophic trafficking, or astrocytic TrkB. T1 is required for exercise-related neuroprotection.

In human studies, RCTs should integrate mechanistic biomarkers with clinically meaningful endpoints such as UPDRS scores, gait speed, 6MWT, balance, fall risk, cognition, mood, sleep quality, fatigue, constipation, and quality of life. Existing studies have begun to connect exercise-related biomarker changes with clinical outcomes, but they do not yet prove that central BDNF/TrkB activation mediates functional improvement. Future trials should therefore combine standardized exercise protocols, repeated biomarker sampling, medication-state control, digital mobility assessment, and, where feasible, neuroimaging or CSF/extracellular vesicle biomarkers.

### 8.4 Bridging landmark studies and precision exercise

Another translational priority is to connect landmark exercise studies with modern biomarker-driven research. Early animal and human studies showed that forced use, treadmill training, and repeated motor activity can improve motor performance, modify DA-related signaling, influence corticomotor excitability, and support activity-dependent plasticity in PD-relevant contexts. These foundational findings remain important because they established that exercise can affect the PD nervous system beyond general fitness, although many were not designed to test BDNF/TrkB signaling with modern pathway-specific methods.

Historically, foundational animal studies by Tillerson and colleagues showed that forced limb use and treadmill exercise could improve behavioral and neurochemical outcomes in 6-OHDA-related PD models, whereas forced nonuse could exacerbate dopaminergic injury (261–263). Work by Fisher, Petzinger, and colleagues further demonstrated that treadmill exercise could promote behavioral recovery, neuroplasticity, and dopaminergic neurotransmission in MPTP-lesioned mice (264, 265). Human studies by Fisher and colleagues and del Olmo and colleagues subsequently suggested that exercise or rhythm-based training may influence corticomotor excitability and brain metabolic activity in patients with PD (266, 267). In addition, Cohen et al. reported that prior forced limb use produced neuroprotective effects in 6-OHDA-treated rats, possibly through GDNF-related mechanisms (268). Together, these landmark studies provided an early rationale for later mechanism-focused research on exercise-induced neuroplasticity, including BDNF/TrkB-related pathways.

Future research should also account for disease heterogeneity. PD varies across motor phenotype, non-motor symptom burden, disease duration, medication response, age, sex, comorbidities, baseline PA, sleep status, cognitive status, and cardiovascular fitness. These factors may influence both exercise tolerance and neurotrophic responsiveness. A mechanism-informed precision exercise framework may therefore be needed, in which exercise modality and dose are matched to individual biological and clinical profiles. Overall, the next stage of research should shift from asking whether exercise increases BDNF to asking how, where, in whom, and under what conditions exercise engages BDNF/TrkB-related neuroprotective mechanisms.

## 9 Conclusion

Exercise is a clinically valuable adjunctive strategy for PD because it can improve motor function, selected non-motor symptoms, and overall functional status while also engaging biological processes relevant to neuroprotection and neuroplasticity. Within this framework, BDNF/TrkB signaling represents a plausible mechanistic link between repeated physical activity and adaptive responses in PD-relevant neural systems.

Current evidence suggests that exercise may influence BDNF/TrkB-related mechanisms across three major domains: neuronal survival, synaptic plasticity, and microenvironmental homeostasis. Preclinical studies provide comparatively direct support for exercise-related modulation of PI3K/Akt–GSK3β and MAPK/ERK–CREB signaling, particularly when pathway activation is reported together with TH preservation, oxidative stress regulation, inflammatory control, and behavioral recovery. By contrast, human studies support the clinical benefits of exercise but rely largely on peripheral BDNF, which should be interpreted as an indirect biomarker rather than direct evidence of central TrkB activation.

Important uncertainties remain regarding TrkB isoform-specific regulation, astrocyte-dependent trophic modulation, PLCγ-related signaling, trophic trafficking, biomarker validity, and exercise prescription–mechanism matching. Therefore, exercise should currently be regarded as a biologically meaningful and clinically useful adjunctive intervention with translational potential, rather than as a proven disease-modifying therapy. Future mechanism-guided studies are needed to determine whether exercise-induced BDNF/TrkB changes are causally linked to durable neuroprotective and functional outcomes in PD.
