# Supplementary material for: Exercise modulation of BDNF/TrkB signaling in Parkinson’s disease: an evidence-calibrated review of neuroprotective mechanisms, biomarker limitations, and translational gaps
Source: Front Neurol. 2026 Jun 25;17:1860227. doi: 10.3389/fneur.2026.1860227 (PMC13345846; doi:10.3389/fneur.2026.1860227)
Supplement: Supplementary file 1 [file Table_1.docx]

| **Supplementary Table 1. Definitions of key terms used in this review and supporting references** | | | |
| --- | --- | --- | --- |
| **Term** | **Definition in this review** | **Relevance to this review** | **Supporting references** |
| Adaptive plasticity | The capacity of neural circuits to undergo beneficial structural or functional modification in response to repeated stimulation, training, or environmental demand. | Used to describe exercise-related changes that may support motor learning, synaptic stability, and functional compensation in PD. | (272–274) |
| Activity-dependent plasticity | Neural modification driven by repeated neuronal activation, sensory input, motor practice, or behavioral experience. | Relevant because exercise provides repeated sensorimotor and metabolic stimulation that may engage BDNF/TrkB-related plasticity. | (274,275) |
| Neuroprotection | Biological processes that preserve neuronal structure or function under pathological stress. | In PD models, this may include preservation of dopaminergic neurons, striatal TH expression, mitochondrial integrity, and synaptic markers. | (43,223) |
| Trophic reserve | The capacity of neurons and their microenvironment to maintain sufficient neurotrophic support under stress. | Used to explain why vulnerable nigrostriatal dopaminergic neurons may be sensitive to impaired BDNF/TrkB signaling. | (43,114) |
| Ligand processing | The conversion of precursor BDNF into mature BDNF through intracellular or extracellular cleavage. | Important because proBDNF and mature BDNF have different biological effects and activate different receptor-related responses. | (276,277) |
| proBDNF | The precursor form of BDNF that preferentially interacts with the p75^NTR^–sortilin complex. | Generally associated with synaptic weakening, structural regression, and pro-apoptotic signaling. | (276,277) |
| Mature BDNF | The cleaved mature form of BDNF that primarily activates full-length TrkB. | Central to BDNF/TrkB-mediated neuronal survival, synaptic plasticity, and exercise-related neurotrophic adaptation. | (277,278) |
| TrkB-FL | Full-length tropomyosin receptor kinase B containing the intracellular tyrosine kinase domain. | The main signaling receptor through which mature BDNF activates PI3K/Akt, MAPK/ERK, and PLCγ pathways. | (278,279) |
| TrkB.T1 | A truncated TrkB isoform lacking the intracellular tyrosine kinase domain; it is enriched in astrocytes and may participate in Ca^2+ regulation, cytoskeletal organization, extracellular homeostasis, and local trophic modulation. | Discussed as an emerging regulator of astrocyte-related BDNF/TrkB signaling in PD and exercise-related neuroprotection. | (108,280) |
| Peripheral BDNF | BDNF measured outside the central nervous system, usually in serum or plasma. Peripheral BDNF is accessible for repeated sampling but is influenced by platelet release, sample processing, inflammatory state, medication exposure, circadian rhythm, age, sex, and physical activity level. | Used in clinical studies as an accessible biomarker, but it should not be interpreted as a direct measure of central BDNF/TrkB activity. | (281–283) |
| Central BDNF/TrkB activity | BDNF/TrkB-related signaling occurring within brain regions such as the substantia nigra, striatum, cortex, or hippocampus. In human studies, it is difficult to measure directly because region-specific brain tissue and molecular readouts from the SNpc or striatum cannot usually be obtained from living participants, whereas serum, plasma, CSF, saliva, tears, or extracellular vesicles provide only indirect and non-region-specific information. | More directly relevant to PD neuroprotection than peripheral BDNF, but difficult to verify in routine human exercise trials. | (43,283,284) |
| Pro-survival signaling | Intracellular signaling that reduces cell-death vulnerability and supports neuronal maintenance. | Mainly discussed in relation to BDNF/TrkB–PI3K/Akt–GSK3β signaling. | (43,278,279) |
| Pro-plasticity signaling | Intracellular signaling that supports synaptic remodeling, dendritic adaptation, and activity-dependent functional change. | Mainly discussed in relation to BDNF/TrkB–MAPK/ERK–CREB signaling. | (273,274,278) |
| Signaling endosome | An internalized receptor–ligand complex capable of transmitting trophic signals from distal neuronal compartments to the soma. | Relevant to long-range trophic communication in nigrostriatal dopaminergic neurons. | (47,285) |
| 6-OHDA model | A toxin-induced PD model using 6-hydroxydopamine to lesion catecholaminergic neurons, especially in the nigrostriatal pathway. | Commonly used to assess dopaminergic terminal loss, striatal TH depletion, rotational behavior, and exercise-related neuroprotection. | (223,286,287) |
| MPTP model | A toxin-induced PD model based on mitochondrial complex I-related dopaminergic toxicity. | Commonly used to examine nigral dopaminergic neuron survival, striatal dopamine depletion, oxidative stress, and exercise-induced molecular adaptation. | (287–289) |
| Tyrosine hydroxylase | The rate-limiting enzyme in dopamine synthesis and a widely used marker of dopaminergic neuron or terminal integrity. | In PD models, reduced TH suggests dopaminergic degeneration, whereas exercise-related TH preservation suggests partial dopaminergic protection. | (290) |
| Exercise prescription–mechanism relationship | The relationship between exercise modality, intensity, frequency, session duration, total intervention length, progression, adherence, and the biological mechanisms engaged by exercise. | Used to emphasize that different exercise paradigms may not activate BDNF/TrkB-related pathways to the same extent. | (207,220,222) |
| Dimerization | The process by which two receptor molecules form a paired receptor complex after ligand binding. | Relevant because mBDNF binding promotes TrkB-FL dimerization, which is required for receptor activation and downstream signaling. | (279,291) |
| Autophosphorylation | The phosphorylation of specific intracellular tyrosine residues within an activated receptor by its own kinase domain. | Relevant because TrkB-FL autophosphorylation initiates downstream PI3K/Akt, MAPK/ERK, and PLCγ signaling. | (278,279,291) |
| Endosome | An intracellular membrane-bound vesicle formed after endocytosis of cell-surface molecules. | Relevant because internalized BDNF–TrkB complexes can form signaling endosomes that support long-range trophic communication. | (47,285,292) |
| Nuclear transcription | The process by which transcriptional regulatory mechanisms in the nucleus initiate gene-expression programs involved in neuronal survival, synaptic maintenance, and plasticity. | Relevant because retrograde BDNF/TrkB signaling can influence transcriptional programs involved in neuronal survival and synaptic plasticity. | (47,278) |
| **Table note.** BDNF, brain-derived neurotrophic factor; TrkB, tropomyosin receptor kinase B; TrkB-FL, full-length tropomyosin receptor kinase B; TrkB.T1, truncated tropomyosin receptor kinase B isoform T1; mBDNF, mature brain-derived neurotrophic factor; proBDNF, precursor brain-derived neurotrophic factor; p75^NTR^, p75 neurotrophin receptor; PD, Parkinson’s disease; SNpc, substantia nigra pars compacta; CSF, cerebrospinal fluid; PI3K/Akt, phosphoinositide 3-kinase/Akt; MAPK/ERK, mitogen-activated protein kinase/extracellular signal-regulated kinase; PLCγ, phospholipase Cγ; CREB, cAMP response element-binding protein; GSK3β, glycogen synthase kinase 3β; TH, tyrosine hydroxylase; DA, dopamine; 6-OHDA, 6-hydroxydopamine; MPTP, 1-methyl-4-phenyl-1,2,3,6-tetrahydropyridine; RCT, randomized controlled trial; PA, physical activity. Reference numbers indicate supporting sources for the definitions used in this review. | | | |
